# A method and approach for evaluating coparenting events during couples group interventions

**DOI:** 10.3389/fpsyg.2024.1463773

**Published:** 2024-10-09

**Authors:** James P. McHale, Karina Irace, Philip Cowan, Carolyn Pape Cowan, Eric Odgaard

**Affiliations:** ^1^Family Study Center, University of South Florida, Tampa, FL, United States; ^2^Department of Psychology, University of California, Berkeley, Berkeley, CA, United States; ^3^Department of Psychology, University of Tampa, Tampa, FL, United States

**Keywords:** coparenting, couple and relationship education (CRE), couples groups, rating system, group dynamics

## Abstract

**Introduction:**

When interventionists stimulate productive father-mother dialogues around coparenting, there are numerous potential benefits for families. Families stand to benefit from more positive involvement of fathers with both coparents and children, key contributors to healthy child developmental outcomes. In this report, we introduce a new strategy and rating system for helping practitioners and supervisors assess the nature and quality of coparenting-related dialogues and conversations in the context of couples group interventions.

**Method:**

The system derives from analysis of 24 relationship-enhancement groups, 13 enrolling English-speaking couples and 11 enrolling Spanish-speaking couples, all parents of young children. All groups were co-led by a male-female team explicitly trained to focus on marital and parenting themes and supervised to address couples issues - not coparenting issues explicitly. All co-leaders spoke the native language of group participants. We documented how frequently coparenting events occurred, and how the nature and quality of events varied within and across groups.

**Results:**

Overall, in both English- and Spanish-speaking groups expressly assembled to focus on marital and parenting issues, coparenting events occurred relatively infrequently. At the same time, both mothers and fathers appeared motivated to raise and discuss issues associated with their coparenting, and extended discussions about coparenting issues broached by the parents blossomed in approximately 37% of all instances. Process-oriented (rather than didactic) co-leader responses appeared especially helpful in scaffolding prolonged coparenting discussions.

**Discussion:**

We propose that use of the system as a training, supervision and self-assessment tool can help clinicians become more consciously aware of how well their interventions succeed in promoting and scaffolding coparenting conversations during group interactions.

## Introduction

Over the past 30 years, two complementary lines of inquiry have helped expand a once-narrow focus on mother–child relationships in the child development literature and enhanced clinical and preventive efforts benefiting families with young children. First, converging evidence from scores of observational studies of coparenting dynamics within diverse family systems have established that children benefit when the adults responsible for their care and upbringing—their coparents (McHale et al., 2022a; McHale et al., 2022b; McHale et al., 2024; McHale and Lindahl, 2011)—work collaboratively as a supportive, coordinated team. Second, unprecedented growth of federal and state-funded programs designed to support healthy marriages and promote responsible fatherhood have given rise to evidence-based interventions for married and committed couples delivered in group settings, guided by curricula designed to strengthen couple partnerships, foster greater father involvement, or both (Halford and Bodenmann, 2013; Hawkins et al., 2008). Group interventions have a sound clinical and empirical base drawing on extensive work by prominent marital researchers (Cowan and Cowan, 1992; Gottman et al., 2010).

To date, however, unexpectedly little attention has been given to the relational dynamics within couples groups pertaining to issues relevant to coparenting. While there have been studies examining fidelity to standardized curricula, such efforts focus largely on whether elements of manualized treatments are delivered with fidelity in the ways that curriculum designers intended, rather than on the extent to which the activities of group leaders and group members elevate and sustain exploration relevant to coparenting conflict and communication *per se* (Ketring et al., 2017). This is a potentially important informational gap, given that more positive coparenting processes in families have both proximal and distal effects on children’s safety, security and socioemotional adjustment (Feinberg, 2003; McHale and Lindahl, 2011).

In the literature on couples group interventions to date, there has also been comparatively less attention given to whether interventions delivered in community settings with diverse clientele have the same positive aftereffects as have been found in studies of middle-class couples seen in university and clinic settings (Hawkins, 2019). This line of work is important, underscored by Urganci et al. (2024) analysis of a large sample of couples (*N* = 1,595) drawn from Parents and Children Together (PACT), a Healthy Marriage and Relationship Education (HMRE) program for low-income couples. In their analysis of PACT baseline data, more than half of couples participating in community based RE programs were experiencing moderate to severe levels of relationship distress and had concerns that their relationship was in trouble. Using the approach taken in the official evaluation of the PACT program (Moore et al., 2018; Urganci et al., 2024) determined that contrary to expectations, there were no significant treatment effects for these couples. They found that more distressed couples were no better off 1 year after receiving RE than couples with similar concerns who did not receive RE. Treatment effects were limited to those couples who entered the program already in happier, more stable relationships.

These findings are not without precedent; there has been a recurring line of thought that the intensive relationship focus of many RE programs is not always the best fit for lower income couples parenting young children. Rather, fathers and mothers in such families may respond more favorably to interventions focused on their child and on their coparenting relationship (McHale et al., 2012; Pruett et al., 2017). There is emerging, albeit still limited evidence that coparenting-themed interventions hold appeal for certain lower-income families (McHale et al., 2022a,b), and that well-conceived programs enrolling lower income families and maintaining a relationship focus can yield desired longer-term benefits. Among the more prominent pioneering studies in this regard has been the Supporting Father Involvement (SFI) project (Cowan et al., 2007).

The SFI program model encourages fathers’ involvement through a coparenting lens with the goal of improving the well-being of family members and strengthening relationships between parents and between parents and children. The original SFI study examined whether group interventions created to support couple relationships and father engagement could also help families at the lower end of the socioeconomic continuum as they have for the middle-class samples most often featured in the research literature. SFI evaluated the effectiveness of an intervention to facilitate the positive involvement of low-income Mexican American and European American fathers with their children, in part by strengthening the men’s relationships with their children’s mothers. The study was a randomized clinical trial in which participants were assigned to a 16-week couples group, a 16-week fathers group, or a single-session control group. Couples in all conditions included partners who were married, cohabiting, and living separately but raising a young child together. Published results from this work have highlighted the efficacy of the groups in promoting relationship quality and father engagement in the manner predicted (Cowan et al., 2014; Cowan et al., 2009; Kline Pruett et al., 2019).

Historically, the question of “what works?” in relationship education has been an interest of marital researchers, though a focus on coparenting within traditional couples group formats has rarely been flagged as a topic for considered exploration. Recently, a line of research spearheaded by clinical family researchers in Switzerland has taken interest in whether intentional interventions targeting coparenting in the context of couples therapy have an impact on coparenting outcomes (Darwiche et al., 2022; Nunes et al., 2022). This work is in its early stages, and has been conceived to test a particular model, but the novel intention of the work is meritorious. There would be parallel value in examining what transpires in couples and relationship education groups, currently the major means of supporting families with young children in the United States, since couples group formats do afford couples an opportunity to attend to issues related to coparenting children while they are together. Unlike the family’s marital subsystem, which is dyadic in nature, coparenting relationships are by definition at least triadic in nature, pertaining to the couples relationship vis a vis one or more referent children (McHale, 2009).

On the one hand, effectively addressing important marital issues such as tolerating difference, problem-solving effectively, and resolving conflict might be expected to bear directly on issues related to the couple’s work together coparenting their children, as some intervention studies have suggested (e.g., Lavner et al., 2019). Indeed, most research studies that have examined marital and coparenting systems in the family separately have verified that there is a significant relationship between functioning in these two distinct family subsystems (Christopher et al., 2015; Favez and Frascarolo, 2013; Feinberg et al., 2016; McHale, 2007). But at the same time, conflict related to children also calls into play a more complex triadic emotional system (McHale et al., 2024). Issues of competition, exclusion, jealousy and other triangular dynamics (Bowen, 1976) can make coparenting problems more challenging to discuss and resolve in a couples group setting than dyadic couples issues such as expressions of affection, handling family finances, and other core marital themes, and hence it is unclear whether such issues are given significant voice when they do come up in couples groups.

To advance the study of coparenting events within couple relationship groups, needed are strategies and tools that can help establish the extent to which such groups - convened as they are to aid couples with marital and parenting issues – actually evoke and accommodate discussions of coparenting themes. This question is a somewhat different one than the question of whether coparenting-themed groups stand as effective alternatives to couples groups. Initial evidence suggests that with middle-income couples, both types of groups can have beneficial effects (Doss et al., 2014). Rather, specific information is needed about the quality, nature and frequency of naturally occurring coparenting exchanges and events as they coalesce between practitioners and parents during the course of couple and relationship-enhancement groups serving fathers and mothers parenting young children.

Discussions of coparenting can be challenging to broker in a group setting as dissonant views between coparents can evoke concerns about uncontained conflict or high emotions. Equally, when interventionists avoid extended discussions about coparenting differences and fail to coax couple and group communication or to explore problem-solving when opportunities arise, they risk signaling to parents that such conversations are chancy and best left unexplored. Since couple and relationship enhancement interventions aspire to enhance better couple communication and problem-solving, a detailing of the nature of coparenting events in couples groups, and identification of how such events blossom – or fail to blossom – when they do surface would be of considerable value both to practitioners conducting couples groups, and to supervisors and training programs working to build the coparenting expertise of less experienced interventionists.

To begin study of this important issue, this report examined couples groups from the original wave of SFI families, exploring the extent to which the groups afforded opportunities for participants to engage in conversations about the family’s coparenting relationship. The original SFI sample is a relevant target for these analyses, because the group leaders who served as interventionists in the original SFI study were supervised to focus on marital and parenting themes, and not coparenting *per se*. The analyses of spontaneously emerging coparenting events hence provided a relevant test of the extent to which well-conceived couples group formats provide a generative platform for coparenting discussions to surface and flourish - if interventionists have been trained principally to focus on couples and their relationships.

To help address this question, we designed a new rating system to identify and characterize coparenting events during couples groups sessions—the frequency with which such occur, how the nature or quality of these events differed from each other, and how different groups varied in their embracing of coparenting events. This tangible means for spotting and documenting coparenting events, successes, and missed opportunities to heighten coparenting awareness and communication competencies during couples groups introduces a needed, value-added contribution that can sharpen the focus of both intervention design and evaluation. Further, the capacity to quantify the nature and quality of coparenting events and exchanges also stands to advance theories of family functioning maintaining that the enhancement of coparenting quality in the family is a key to fostering young children’s development and adjustment.

### Research questions

1. How often do coparenting events occur during couples group interventions designed to strengthen relationships in families with young children?
2. What is the character of these events, both within and across different groups?
3. Are there specific elements of group process that distinguish groups in which coparenting becomes a more prominent focus from those in which coparenting is less prominent?

## Method

### Participants

Participants were enrolled in the “Supporting Father Involvement” (SFI) project, a preventive intervention designed to examine the effectiveness of couples groups for promoting father involvement in low-income families sponsored by a state Office of Child Abuse Prevention. The SFI project and staff were located within Family Resource Centers in four California counties (San Luis Obispo, Santa Cruz, Tulare, and Yuba) in primarily rural, agricultural, low-income communities with a high proportion of Mexican American residents. At each site, project staff recruited some participants through direct referrals from within the Family Resource Centers and most participants from other county service agencies, talks at community organizational meetings, ads in the local media, local family fun days, and information tables placed strategically at sports events, malls, and other community public events where fathers were in attendance. Because the project was conceptualized as preventive—to help families early in the family formation years before smaller problems become intractable—the project targeted expectant parents and those with a youngest child from birth to age 12.

During the recruitment and screening process, parents had to meet the following additional criteria: (a) both partners had to agree to participate; (b) both father and mother had to be the biological parents of their youngest child and raising the child together, regardless of whether they were married, cohabiting, or living separately; and (c) neither parent suffered from a mental illness or drug or alcohol abuse problems that interfered with their daily functioning at work or in caring for their children. Finally (d) couples were not accepted into the study if there was a current open child or spousal protection case with Child Protective Services or an instance within the past year of spousal violence or child abuse.

Of the 276 couples who completed pre-test and post-test assessments and completed at least one group meeting, just over two thirds of the participants (67%) were Mexican American, 27% were European American, and 6% were Asian American, African American, Native American, or mixed race. On entering the study, 72% of the couples were married and living together, 22% were cohabiting, and 6% were living separately and raising a child together (separated, divorced, or never-married, never cohabiting couples). Participants were not screened for income, although the sample was heavily weighted toward low incomes. Median household income was $29,700 per year, with more than two thirds of the sample falling below twice the federal poverty line at that time ($40,000 yearly household income for a family of four). 2.5% had household incomes over $100,000 per year. A large majority (79%) of the fathers and a minority (39%) of the mothers had worked for pay during the week prior to their baseline assessment. About half of the participants had completed high school or beyond. At baseline, the number of children in the household ranged from 0 (mother was pregnant with a first child) to 7, with a mean of 2.34 children; the median age of the youngest child was 2.25 years.

For this report, we analyzed all 24 couples groups from the original SFI study, each of which enrolled 4 to 5 couples. 54% of the groups analyzed consisted of English-speaking couples and co-leaders and 46% were comprised of Spanish-speaking couples and co-leaders. The thirteen English-speaking groups were mostly constituted by European and Mexican American parents with a smaller proportion of African- and Asian-American parents. The eleven Spanish-speaking groups, by contrast, were constituted only by Mexican and Mexican American parents. Hereafter, we will use the terms “English-speaking groups” and “Spanish-speaking groups” only to respect the diversity and complexity in ethnicity that both groups represented.

### Design and procedure

All procedures were approved by the University of California at Berkeley’s Institutional Review Board. Consent forms included permission to use participants’ responses to questionnaires and video recordings for research purposes. The video cameras were visible in the meeting room. All groups were led by male–female pairs of mental health professionals selected by project directors based on clinical expertise, training, and experience with couples or groups or both, knowledge of family and child development, cultural fluency and sensitivity, and the ability to work collaboratively with other professionals and agencies.

The original study design consisted of three different conditions, determined by random assignment: a 16-week group for fathers, a 16-week group for couples, and a low-dose comparison condition in which both parents attend one 3-h group session. All interventions were led by the same trained mental health professionals who focused on the importance of fathers to their children’s development and well-being. The one-time meeting and the 16-week curricula for fathers and couples’ groups were based on an evidence-based five-domain family risk model of the central factors that research has shown are associated with fathers’ positive involvement with their children (Cowan and Cowan, 2012): (a) individual family members’ mental health and psychological distress; (b) the patterns of both couple and parent–child relationships transmitted across the generations from grandparents to parents to children; (c) the quality of the relationship between the parents, including communication styles, conflict resolution, problem-solving styles, and emotion regulation; (d) the quality of the mother–child and father-child relationships; and (e) the balance between life stressors and social supports outside the immediate family.

The groups were formed by 6 to 12 fathers or five to nine couples; they met for 2 h each week for 16 weeks and all sessions were videotaped. The curriculum was designed in a semi-structured fashion. Sessions included exercises, structured discussions, and short presentations together with an open-ended time during which participants were invited to raise their real-life issues and concerns for discussion and problem solving. Each SFI session was devoted to coverage of at least one of the five main domains of the curriculum. The couples and the fathers-only curricula were comparable, and almost identical, covering the same topics in the same order. The teaching segments about individual, couple, and parenting issues were identical. The exercises for the individual, parenting, and life stress topics were also identical. The only difference came in the sessions addressing couple relationships, in which fathers described their couple issues and were encouraged to do “homework” in which they explored these issues with their partner in between group meetings.

Based on the topical themes, we decided to observe two sessions for our analysis – one in which the primary theme for the week was to be devoted to a discussion of parenting styles and the other in which the theme was to be devoted to the division of labor. Our choice of these two specific sessions was guided by collective clinical experience that parenting and the division of childcare labor can be especially evocative topics for coparents (Pruett, 2010).

During the first year of the project, the first two authors (JM, KI) watched the videotapes both independently and together and once a system had been developed and categories reliably identified and coded, met together with the third and fourth authors (PC and CC) to review and discuss a series of the coparenting events that had been identified. During this second stage of the work the investigators reviewed the system, categorized events, and made decisions about how to identify stop and end points for “bounded units.” A bounded unit was an event that started with a statement by a speaker (either a parent or group leader) that could be considered a coparenting-related bid, prompt, or query, and that ended once a subsequent speaker’s comment ended the focus on coparenting by effectively shifting the conversation in a different direction. Once this development process was completed, the tapes were evaluated by the second author and a second trained coder. After a period of initial training during which three cases were rated independently and discussed together, these two individuals evaluated all 48 sessions for the 24 couples groups. The second author (a native Spanish-speaking coder) rated events for the Spanish-speaking groups and the trained coder rated events in the English-speaking groups.

### Description of the coding process

In reviewing videotapes for each session, coders identified and characterized all “coparenting events” that emerged during the group. A coparenting event was defined as a bounded unit relating specifically to the two parenting individuals’ perspectives on or about their shared child. Common events included expression of an opinion about the child or about parenting, whether the opinion was shared (or not) by the coparent, and remarks comparing how the two parents handled things with their child – whether similarly or differently - as individuals.

Each bounded coparenting unit involved the person or couple who raised the issue. The bounded unit could also involve group leaders and/or members of the group, if they spoke up while the coparenting event was underway. Using structured coding sheets, coders systematically took note of whether each target event was preceded and triggered by a group leader prompt, evolved spontaneously, or began when a group leader explicitly followed a parent’s comment about their child or about parenting by asking the other parent if s/he saw things the same way. These latter events, while rare, transformed an event that might otherwise have been understood as one individual’s unique personal standpoint about parenting into a coparenting event. They occurred when a group leader saw potential for a family-specific coparenting conversation and prompted further consideration of the topic by the same person and couple who brought up the issue. By contrast, events coded as having been triggered by a group leader prompt typically either (a) followed a question that had been posed to the group as a whole or (b) followed a question asked to certain individuals in the group, but without engaging the coparent. Spontaneously evolving coparenting events were always initiated by a member of the group, with no prompting.

Whenever an event was identified that met the preceding criteria, coders reviewed the tapes several times to be able to specify precisely when the event began and ended, and recorded verbatim all statements that followed, and specifically related, to the initiating comment of the individual who triggered the event. Raters recorded several additional units of information (see below) and then assigned one of 10 different codes to capture the quality of the events.

### Measures

This coding process yielded frequency data for each of the following items:

- The total time subsumed by each event – shorter events signifying topical conversations that may have had potential, but did not blossom, and longer events including conversations that involved deeper exploration and/or multiple speakers.
- The partner who initiated the event (mother or father).
- The spontaneity of the initiating partner’s comment (i.e., whether it was made as a direct response to an explicit group leader prompt related to coparenting issues, or whether the mother or father raised the issue on their own)
- Whether the partner of the person who initiated the issue joined in on the exchange their spouse or partner had initiated.
- Whether a group leader responded to the coparenting issue that was raised by the parent.
- Whether other wives and husbands in the group responded to the coparenting conversation.
- How involved each person remained (how many additional comments they made) until the event wound to a close (as determined by a lasting topic shift).

Once all these features had been recorded for each given event, raters assigned one of ten codes (most with sub-codes) to capture the overall quality of the event. The system was designed so that lower-end scores reflected coparenting monologues or brief dialogues with negligible contribution by/payoff for others in group. That is, low-end scores were used to denote events that had the potential to blossom into a prolonged exchange on the topic of coparenting but did not. Why they did not could be attributed to one or more reasons. For example, the speaker’s initiating comment may not have been responded to by their partner, by group members and/or by co-leaders at all. Or the response they received to their initiating comment shifted the conversation away from coparenting and into some other area (child behavior, parenting styles, stress management). All low-end scores, however, shared the characteristic that what could have been a coparenting-related discussion never got going, having been squelched in some way. Events receiving higher scores played out for a longer period, involved the partner and/or others in the group, and (when at their best), resulted in a productive resolution or insight for both partners that were witnessed and sometimes shared in by others in the group.

## Results

The Results section is divided into three parts. In the first we provide a summary of the new system that identified and characterized coparenting-related events during the couples groups. This first section recapitulates each category, from comments never responded to by partners, group members or leaders through the extended and very productive discussions having everyone involved. We describe the overall “lay of the land” in terms of how frequently each category event occurred, and include excerpts taken from the groups that illustrate different categories. The second section provides a global look at the contributions of group leaders and of group members in their different group roles. Finally, we present a quantitative analysis of different interior processes among the 24 groups with respect to the quality of coparenting events within those groups.

### Quality of the 198 bounded coparenting units identified across the 24 couples groups

The 10 codes developed for the system are presented in Table 1, along with their frequency and their total time of occurrence (in minutes) during the 24 groups. As detailed further below, we divided the categories into conceptual groupings, with categories 0–5 capturing events that by and large did not blossom into meaningful or extended considerations of the topic raised, and categories 6–10 capturing more protracted and potentially helpful explorations. Below, we describe each category and provide a few examples to illustrate events that received these rating scores.

**Table 1:** Frequency and duration of various categories of coparenting events.

Codes	Frequencies		Definition
	*n*	Time	
			*Unsuccessful group leaders actions*
0a	0	0:00:00	Failed process-oriented intervention by leaders to transform a parenting comment to a coparenting event.
0b	0	0:00:00	Failed spontaneous leader comment in trying to open a coparenting dialogue.
0c	3	0:03:01	Failed didactic intervention by leaders to transform a parenting comment into a coparenting event
Totals	3	0:03:01	
			*Missed opportunities*
1a	28	0:20:55	A parent’s coparenting comment that fizzled because neither the partner nor the group picked up on the coparenting bid.
1b	9	0:06:50	Equal to 1a, but the parent’s comment was in response to a previous leader’s coparenting bid.
2a	2	0:05:13	A parent’s coparenting comment triggered at least a related comment by another group leader.
2b	15	0:29:36	A parent’s coparenting comment triggered at least a related comment by another group leader.
Totals	54	1:02:34	
			*Brief, relevant dialogues without meaningful payoff*
3a	27	0:36:44	A coparenting dialogue between partners went unnoticed and hence not responded to by leaders/others in the group.
3b	14	0:13:51	Parallel to 3a, except the partners’ dialogue was in response to a previous leader’s coparenting bid.
4	23	0:23:54	Parents’ dialogue/monologue responded to by leaders with a re-statement/acknowledgment of the coparenting issue.
5	3	0:06:26	A partners’ dialogue responded to by group members without leader intervention.
Totals	67	1:20:55	
			*Brief, relevant dialogues with some minor payoff*
6a	1	0:01:39	A parent’s coparenting comment that did not trigger his/her partner but is responded to by group members.
6b	7	0:11:59	Leaders’ comment in response to a parent’s coparenting comment that did not trigger the partner, but that triggered group member(s).
7a	7	0:08:12	A coparenting dialogue between partners that went well with no intervention by leaders.
7b	17	0:39:13	A coparenting dialogue between partners punctuated by a specific leader’s comment, but nothing further.
Total	32	1:01:03	
			*Brief, prolonged relevant dialogues with useful payoff*
8a	15	0:47:09	Leaders posed strategic questions to amplify a couple’s issue; they paid attention to the couple, but without resolution.
8b	6	0:14:48	Equal to 8a but achieving some resolution.
9a	14	1:25:10	Leaders’ attention to a couple’s issue reached a payoff for the group, but failed to finish the original couple’s issue.
9b	5	0:17:52	A couple’s issue reached a payoff for the group, triggering active group participation, failing to finish the central couple issue.
10	2	0:14:27	The issue reached payoff for both the couple and the group.
Total	42	2:59:26	
Grand Total	198	6:26:59	

### Category 0: group leader attempts to evoke coparenting-related discussion; parents do not respond (1.4% of all events identified)

A relatively small (1.4%) proportion of all coparenting events took the form of a failed attempt by a group leader to prompt the group to consider a coparenting issue. Such attempts were typically generic remarks concerning the importance of coparenting solidarity and teamwork. Codes of 0 were assigned if such comments appeared to be ignored altogether by group members, who instead responded by shifting focus onto a different, non-coparenting-related issue. The proportion of 0 events among the different groups ranged from 0.0 to 0.20 (i.e., 20% of all coparenting events that transpired in the group received codes of 0). Though few were detected, we believe that such events are not uncommon in work with couples – interventionists believe they see a “teachable moment” and so attempt to influence couples by educating them about a coparenting-related topic, only to be met by immediate parental movement onto a different issue.

### Category 1 to 2: parent monologues about coparenting followed by partner/leader/group member non-response and topic shifts (23.86%)

1a: Opportunity for a coparenting dialogue missed because neither the partner nor the group leader picks up on the bid. 13.0% of all coparenting events involved a coparenting concern spontaneously raised by a parent that did not progress further because the initiator’s bid was not responded to further by the partner, group members and /or group leaders. The proportion of 1a events among groups ranged from 0.0 to 0.43. The following is a prototypical 1a event drawn from one of the sessions:

A father commented, “When Tony does not want to eat, I say, ‘Eat your food or go to your room’. And if he cries, he has to go to his room. (I say -) ‘Which one do you chose?’ Then if he starts throwing a fit, I stick with that. If he still cries and throws his fit, then he goes to his room. Then I’ll come back and talk to him, ask him ‘Are you ready to come out?’ or something like that. The more consistent I am with that when it does happen, the more he’ll say ‘Sure I will sit down.’ But then if it does not happen for a few days or I’m not there during dinnertime or something, he just cries and cries and cries. Then we have to do it again, but after two or three times he can see he knows we mean business. And it seems to work good.” The group leader’s response to this father’s story was “Kids need containment, when they have too many choices they can kind of pick whatever they want; sometimes it can be really overwhelming for kids. And so structuring it down, saying ‘you can do this, or this,’ sometimes is really helpful for them. Just cognitively, I do not care how smart they are. They need smaller choices.

Although this father’s story might simply be construed as his own perspective on parenting, it was his indirect mention of problems with inconsistency when he was not at home (and presumably his partner was) that transformed the story into an event that might be considered to involve covert coparenting dynamics. Discussing covert coparenting, McHale (1997) noted, “what happens during alone, one-on-one time with the child may be as or more important in establishing a sense of coparental alliance and authority for the child as what happens when the partners are parenting together” (p. 207). In this Category 1a event, the father shared a concern that if he wasn’t physically present to reinforce his strategy, all his hard-won progress with the son would take a step backward. Moreover, his remark invites an interpretation that his wife chose not to support his efforts when he wasn’t present. However, rather than picking up on this bid and inviting a dialogue (either with the couple, or with the group) about the relevance of coparental support of partner interventions with children, the leaders instead chose to educate the group on the importance of containment for children (i.e., providing psychoeducation about parenting) - and hence a coparenting dialogue never blossomed. We believe that these kinds of events may be of particular interest to interventionists, whose first impulse may often be to educate rather than to deliberately invite and give voice to a potentially contentious discussion of differences about parenting.

1b: 3.2% of all coparenting events involved a coparenting question or comment voiced by one parent that, just as in 1a, was not picked up on and embellished. The only distinction between 1a and 1b was that the initiating parent’s contribution had been activated by a group leader question or comment. However, just as in 1a, the parent’s comment did not blossom into a coparenting dialogue between the speaker and his/her partner because it was not recognized and responded to by the partner, by group members and/or by the group leaders who had prompted the comment. The range of the 1b events among the groups was 0.0 to 0.17.

2a: Another 0.5% of all coparenting events were coparenting monologues that did not materialize into a dialogue between spouses, but that did trigger at least one related comment by another group member. The proportion of 2a events among the groups ranged from 0.0 to 0.06.

2b: Finally, closing out Category 0 to 2, 7.2% of all coparenting events were opportunities for coparenting dialogues that did not materialize between partners but that triggered a related coparenting speech by the leaders. These speeches were like those in Category 0 in that they were psychoeducational interventions. However, they differed from 0 events in two ways. First, they followed a parent’s remark. Second, they included advice, personal experiences and didactic comments about coparenting. The proportion of 2b events among groups ranged from 0.0 to 0.43.

### Category 3 to 5: brief, contained dialogue about coparenting; negligible contribution by/payoff for others in group (34.08%)

3a: Brief coparenting dialogue between parents (2 turns or more) that goes unnoticed or unresponded to by group leaders or others in the group. Of special note, a fairly high proportion of all coparenting events (12.6%) were short coparenting exchanges that emerged spontaneously between parents (2 turns or more) - but went unnoticed or unresponded to by group members and/or group leaders. In such instances, leaders and other group members either missed the exchange altogether or redirected the conversation to a non-coparenting-related topic. The range of the 3a events among the groups was 0.0 to 0.50. As with Category 1a, we believe that Category 3a is of special interest both to interventionists who lead couples groups and to those who work individually with coparents. The following example is prototypical of this category:

A husband, talking about different parenting styles for younger children and teenagers, expressed his belief that parents must be more rigid with younger children than with teens. A group leader replied: “It sounds like you start a little tighter, and when they start to grow up you loosen up.” He says: “yes, I think so.” His wife replied: “I am the opposite. At some point you have to say ‘Absolutely not’ … (feigning a teen’s voice): ‘Mom and Dad, can I go to the party?’… (Then taking a parental voice): ‘No - over my dead body’” Her husband tried to interject, but she spoke over him to continue explaining her position “That is just a flat out ‘no’ - there is not going to be a discussion about it.” In response, rather than turning to the husband to determine what he had tried to interject - or whether his stance did differ from that of his wife - the group leaders instead educate the group about what authoritative parenting is, and how an authoritative parent might respond in this hypothetical case. The flow of the group hence moved away from coparenting, and back to parenting behavior.

This example differs from Code 1a above in that the event of interest actually involved an exchange between the two partners rather than a monologue by one parent that was not picked up upon by anyone else in the group. The mother clearly delineated a difference between herself and her husband (“I am the opposite”). However, the difference between the two never became a thrust of the conversation that followed, in part because of the inaction of the group leaders.

3b: In a related 7.3% of all coparenting events the coparenting dialogue between parents that ended without comment was one that had actually been prompted by a group leader question or comment. However, just as in Category 3a both the group leaders and the other group members missed the opportunity to advance or prolong the coparenting discussion further. Again, most prototypically, the discussion was instead redirected onto a non-coparenting-related topic. The proportion of 3b events among the groups ranged from 0.0 to 0.33.

4: Dialogue responded to, then ended, by leaders with a simple restatement/ acknowledgment of the coparenting issue. 12.8% of all coparenting events involved a brief coparenting dialogue or coparenting-relevant monologue that was responded to by a group leader, who provided either a re-statement of what the speaker(s) said or a perfunctory acknowledgment of the issues. But the event then ended, and there was no further dialogue with either partner or discussion in the group about the issue that had been raised. The proportion of 4 events among the groups ranged from 0 to 1.0 (i.e., all coparenting events that transpired in the group were of this form).

5: Coparenting dialogue responded to by group members with empathic concerns, but without further development in the group. 1.36% of all coparenting events were brief coparenting exchanges between partners that triggered one or more related comments by other group members. While the group member comment(s) could have been offered in empathy, the event then ended; there was no further development of coparenting-related discussion in the group about the issue that had been raised. The range of events coded 5 among the groups was 0.0 to 0.20. Following is a verbatim transcription of a Category 5 event, in which the conversation revealed an ongoing dispute between parents about clothing they chose to put on their children to go out:

Husband B said (ostensibly to Wife C, who had made a comment about getting her son dressed): “Does he…does it matter to you if he matches…?”

Wife B added: “Like if they are going to a birthday party— (and in an apparent aside to her husband)—put that fact out there…”

Wife C replied “Well, if it is important to my partner. He can be hard on me - he’ll be like, *‘he is going to school…*’.”

Female leader said, “Having issues when dressing the child…”

Husband B said, “If my child wants to wear something…”

Wife B said, “We do that during the day, but I do not want to….”

Husband B said: “You know, she is 4 years old. If she wants to wear something, I am glad she wears it. She (referring to his wife) on the other hand, will not go along…And I say, ‘*honey, she is 4 years old’*.”

Male leader said: “If she is okay…?”

Husband B said: “It’s like to me…‘okay honey’.”

Wife B said: “It wasn’t the dress. It was a birthday party, and these patterns…these were different colors. During the day in the house, she can wear what she wants, I do not care but if we were going to…I want….”

Husband A said: “I kind of…where we go, they can wear what they want to wear.”

Wife A said: “No, no, no. Dude, they are going outside the house. No, no.”

Husband A said, “I generally say, wear you want to wear, then they pick it out and come up with something completely absurd. I am more like ‘are they suitable to go outside than actually how they look’. I am not too concerned with looks as long as they are happy.”

Male leader said: “When you think about taking the child outside, it is a reflection of us.”

Husband A said: “Yes.”

Male leader commented that his wife thought differently than he did about their daughter.

Female leader said: “It’s sort of cute….”

Husband B said: “That would not be the reason for me doing that. The reason for me doing that is that she wants to wear that.”

Wife B said: “We’ve seen kids in the store that their parents…I would not do that …if it just for a birthday party, kids play, they get on the ground…I just want the colors to match.”

Female leader (shifting the topic to division of labor) said: “So you do more of the child’s dressing?”

Wife B said: “No, actually, we do it equally.”

This event was interesting both in terms of how it started and the group dynamic that followed. When the husband initiated the conversation by ostensibly addressing a question to a female member in the group about whether matching her child’s clothes mattered to her, he did so with the apparent intention of infusing into the group a discussion he had already had independently with his wife. He appeared to be looking for allies and succeeded in finding one and having his opinion validated when another husband in the group agreed with him. His wife also received support from another female member, such that the central couple’s discussion ultimately ran across gendered lines. Gendered perspectives in couples groups have been discussed by Feld (2003) as one useful means for helping individuals to find validation and support from others of the same gender in their group. She posits that such events occur in a second phase in the development of groups that she calls “the working group”—a subsequent phase to an initial “holding-containing” phase. Working groups, Feld notes, are characterized by the formation of subgroups different than the couple – the most common of which runs across gender lines. Feld cautions that therapists be careful in not to get drawn into any particular “sides” but rather aim to help each subgroup listen to and begin to understand the others.

In the featured scenario, the leaders did not quite manage to do so; the male leader sought to validate the wife’s opinion when he said, “When you think about taking the child outside, it is a reflection of us.” Though his intention was to make the wife feel better, taking a side did not facilitate fathers and mothers in the group’s understanding and accepting of their different positions, or of how their differences might affect their solidarity in the coparental alliance. The event was ultimately given a Category 5 code owing to the husband’s recruitment of allies in the group to validate his opinion. What did not get developed as a coparenting theme was how validation of his opinion discredited and perhaps undermined his wife’s perspective. The differences across gender sides might have been framed and developed further as a metaphor for understanding women and men’s equally legitimate points of view as parents, and for helping the couples develop greater empathy about and support for one another’s perspectives about their children.

### Category 6 to 7: brief monologues/dialogues about coparenting with some minor contribution by/payoff for others in group (18.3%)

6a: 0.3% of all coparenting events were opportunities for coparenting exchanges that failed to materialize between the initiating speaker and his/her partner, but that triggered a coparenting-related conversation among other group members. The proportion of 6a events among groups ranged from 0.0 to 0.03.

6b: In 3.2% of all coparenting events, group leaders responded to one person’s initiating coparenting comment by posing a question or comment to prompt a coparenting dialogue between them and their partner. Though the intervention was unsuccessful in eliciting such a dialogue between partners, it did trigger a coparenting-related monologue or conversation involving other group members. The proportion of 6b events among groups ranged from 0.0 to 0.25.

7a: 3.5% of all coparenting events were brief coparenting exchanges between partners that went well with no intervention (i.e., each partner offered measured counterpoint/ acknowledgement/validation/support). The event then ended with no further response from leaders/group members. The range of the 7a events among the groups was 0.0 to 0.33.

7b: 11.3% of all coparenting events were brief coparenting exchanges between partners that were punctuated by group leaders who commented specifically about the coparenting issue the couple had aired. The event then ended; there was no further dialogue with either partner or discussion in the group about the issues the couple had raised. The range of the 7b events among the groups was 0.0 to 1.0.

Category 8 to 10: Brief or prolonged dialogues about coparenting; significant leader involvement; significant contribution by/payoff for others in group (22%).

8a: In 7.2% of all coparenting events, group leaders attended to the couple’s issue, posed strategic questions that amplified the issue, and enabled productive dialogues about differences. The events, while productive, ended without specific resolution for the couples of the issues they had raised. The proportion of 8a events among groups ranged from 0.0 to 0.40.

8b: In another 3.86% of all coparenting events, group leaders attended to the issue, posed strategic questions amplifying the issue, enabled productive dialogue about differences, and coaxed some resolution (e.g., some evidence that one partner understood/validated the other’s point of view). The range of the 8b events among the groups was 0.0 to 0.25.

9a: 8.31% of all coparenting events were coparenting dialogues between partners responded to by group leaders who prolonged and amplified the coparenting discussion by involving other couples. In these instances, however, the events, while productive for the group, ended without any specific resolution for the couple of the issue they had raised. The range of the 9a events among the groups was 0.0 to 0.50.

9b: 2.16% of all coparenting events were coparenting dialogues between partners that triggered related coparenting comments by other group members. The group discussion, later joined as well by the leaders, prolonged and amplified the coparenting discussion. However as in 9a, the events, while productive for the group, ended without specific resolution for the couple of the issues they had raised. The range of the 9b events among the groups was 0.0 to 0.20.

10 0.8% of all coparenting events reached payoff for both couple and group. The coparenting dialogues between partners were responded to by group leaders who successfully prolonged and amplified the coparenting discussion *and* expanded it to other couples without changing or diluting the issue raised by the original couple. The range of the 10 events among the groups was 0.0 to 0.14.

Following is a verbatim transcription of a Category 9 event, in which group leaders amplified a coparenting dispute about childcare inequities and differences by intentionally inviting other group members to engage in the conversation:

The following is an example of a prolonged coparenting exchange (rated a 9b) that illustrates effects of amplification following a group leader’s well-timed invitation to fathers in the group:

Wife B said: “It seems what I’m trying to say to him because right now I’m in maternity, but I used to be working or doing school, with the kids - and then he came in. I used to be a single parent. For the last 3 years, I am trying to work him into it, and he is….”

Husband B said: “No - get this. This is what a female does. All right…whatever…they can get in trouble.”

Female leader said: “I just want to point out that you are sitting between two women….” (group members laugh at the leader’s joke.)

Husband B continues: “They’ll get on them… and then – no -even just 5 s later: ‘oh it’s okay. Do you want a piece of candy? What do you want?”

Wife B replied: “This is what it is like, especially since X was born, I have a 3 years old screaming: ‘I want my daddy; I want my daddy!’ Who is always gone.”

Husband B said something inaudible.

Female leader said: “Oh!”

Wife B said: “I have a 1 year old on my legs, the dishwasher is going, the TV, you sitting there, the baby crying, he needs to be fed, and the toys need to be picked up. How am I going to do it? I have two hands.”

Husband A said: “I know I can read pretty well…”

Male leader interrupted this comment and said: “I want to hear from a father about what mom said. What did you hear mom say?”

Husband C said: “The same as I hear everyday… blah, blah, blah.”

Husband A said: “I think she has a good point.”

Wife B said: “She is frustrated.”

Husband A said: “I played that role too; a stay home husband with a wife that has to work. I spent the first 2 h after she went to work, and I have a baby too, you know, cleaning the house… the bath, the kid, the dishes, it never stops.”

Wife B began to ask: “How many…?

Husband A said: “And I realized that too. I need to be more flexible when is about to help, but a lot of us, for me, I took it from granted… to take care of the house, the laundry, the kids when you have one person to worry about the baby and yourself, is pretty simple. When we are talking about the kid… men, *I cannot relate very well*. Honestly for me, *I cannot understand a crying kid*.”

Wife B said: “To discipline a kid is…You know, hold the baby for a minute, you know it just has worked.”

Husband B said: “How do I get the kids to be quiet, though?”

Wife B said: “You yell at them.”

Husband B said: “I yell them? I send them to their room.”

Wife B said: “And you shut the door.”

Husband B said: “I shut the door and then, they turn on a movie, and they both sit and they watch it in their room.”

Female leader said: “So, they are like self-parents. If they are watching a movie, they can figure out how to calm themselves.”

Wife D said: “I do daycare…If I ask them to calm down, like on Mother’s Day, I read them a story and it was good.”

This event was instructive in that the leader’s comment simultaneously interrupted, momentarily, an escalating dispute between the coparenting couple, containing mounting tensión that was apparent to group members, and drew other group members in to participate in a consideration of the dissonance being aired. At the start of this event, the mother who voiced the issue lamented her coparenting partner’s lack of support with child care labor, later pivoting to his abrupt manner when disciplining the children. Her coparent, for his part, responded to her critiques by framing their differences as contention between men and women. At the point of the event’s initiation, the mother noted (with a blend of anguish and anger) her struggle to include her partner as a coparent. She recalled managing her single parent role adequately, with her coparenting partner having been the cause of the problems since *he came in*. The portrayal of her encounter and relationship with her partner as having been with someone that *came in* to her life hinted at some distancing of responsibility for personal choices. The dialogue between partners remained tense while featuring two common arguments: inequity in childcare, and disagreements about the coparent’s style of dealing with children. The male leader’s intervention mitigated the increasing emotional strain felt not just by the couple but by the entire group, inviting other fathers to listen to the mothers’ complaints. This opened the discussion to all group members, most pointedly the male subgroup, inviting them to listen empathetically to the female subgroup. This turn of events elevated the quality of the coparenting conversation in the group to a 9 code.

The first father who responded aligned with the father “on the hot seat” to support his expression of the feelings of a man being critiqued by a woman. The second father offered an empathetic response validating the mother’s feelings. The event hence became more productive for the group, but it ended without resolution for the couple who raised the issue. Additionally, the feelings of the father who expressed difficulties dealing with the children when they misbehaved were not validated by any of the women in the group. Rather, they were countered by the mother, and her remarks rekindled the argument anew. Had the leader (or co-leader) expanded the intervention strategy by inviting women in the group to empathically listen to the fathers’ complaints—“what did you hear dad say?”—a strategy encouraged by Feld and Urman-Klein (1993), the group as well as the couple might have found resolution or at least greater understanding of each other’s perspectives. Because this did not happen, only the women’s “side” found some validation. Though this could have helped mothers feel more supported in the group, it also risked reifying a narrative wherein mothers are usually right and fathers usually wrong in the childrearing domain. Such a perspective can sabotage coparenting solidarity in the couple, creating a divide that erodes both marital and coparenting dynamics. Emphasizing complementarity of the coparental relationship (Minuchin and Fishman, 1981) when addressing childrearing differences avoids mother vs. father and women vs. men traps, allowing each coparent to consider how his or her own behavior may prompt or even reinforce unwanted behavior from the other. These things said, the leader’s deliberate interruption and expansion of the coparenting conversation enabled group members to consider looking at concerns and disputes from an alternate perspective.

### Overall contributions made by group leaders, and by coparents in their different group roles

**Figure 1:**
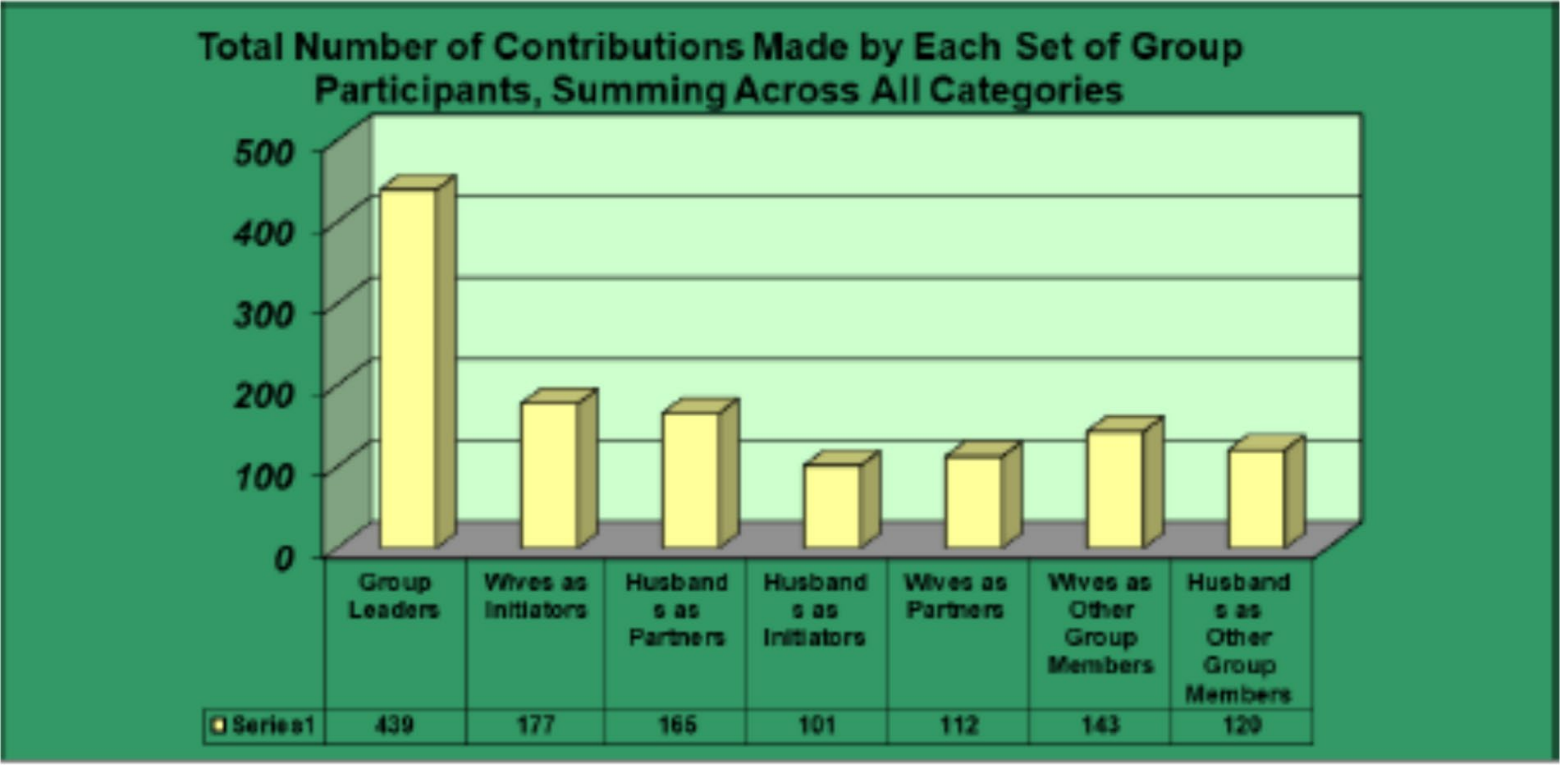
Total contributions by facilitators and by husbands and wives in group roles.

Figure 1 depicts the overall number of contributions made by group leaders, and by husbands and wives across groups in their different participatory roles.

### Analysis of group processes and group differences

**Figure 2:**
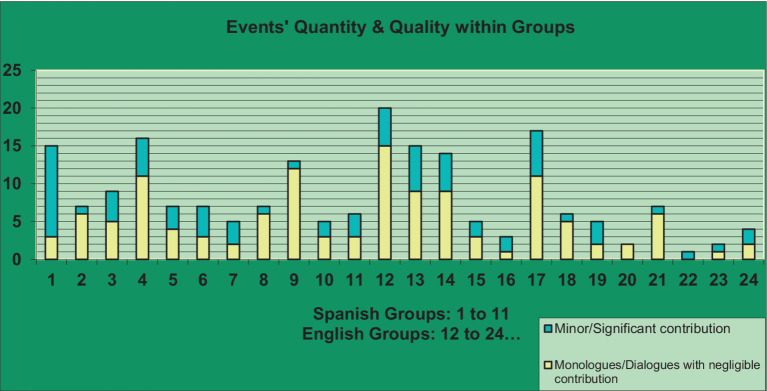
Coparenting event quantity and quality within each of the 24 couples groups.

This section describes conceptually interesting distinctions among the different groups with respect to the coparenting data. First, we graphically illustrate the overall landscape of coparenting events in the 24 different groups. Figure 1 summarizes the proportion of different kinds of events within each group. In Figure 2, we depict events categorized as monologues or dialogues with negligible contribution (categories 0–5) in yellow and events with minor to significant contribution (6–10) in blue.

We then undertook a set of comparative analyses^1^—first examining whether there were any noteworthy differences as a function of linguistic composition of the groups, and then delving into differences among the 24 different groups in the saturation of coparenting content within group conversations.

### Language differences

Overall, group sessions for Spanish-language groups (groups 1–11 in Figure 1) ran a bit longer. This was true for both the Parenting session (*M* = 109.52 min, *S* = 24.38, for Spanish-language groups; *M* = 74.23 min, *S* = 38.68 for English-language groups; *F* (1, 22) = 6.83, *p* = 0.02, η^2^ = 0.24, 95% CI [0.01, 0.48]) and the “Who Does What” session (M = 105.94 min, S = 42.05 for Spanish-language groups compared with M = 62.23, S = 29.57 for English-language group; F (1, 22) = 8.89, *p* < 0.01, η^2^ = 0.29, 95% CI [0.03, 0.52]). We believe this reflected a difference in tempo; many Spanish-speaking groups took an unhurried approach in warming up to each topic gradually, pondering each issue raised. However, virtually all two-hour sessions for both groups stayed focused on the Parenting or Who Does What topic of the day. The only other significant difference between linguistic groups was that during WDW sessions, English-language group leaders made more bids to start coparenting dialogues (Median = 2.00, IQR[1.00, 3.00]) than did the Spanish-language group leaders (Median = 0.00, IQR[0.00, 1.00]); U = 34.50, z = −2.23, *p* = 0.03, r^2^ = 0.21, 95% CI [0.01, 0.53]. It appeared this difference reflected some English-speaking leaders having asked each participant to call out numerical ratings they had given for specific Who Does What survey items. By contrast, most Spanish-speaking leaders did not do item-by-item checks, instead asking what differences coparenting partners saw in how they perceived their contributions to division of labor. Differences as a function of the language in which sessions were conducted by the multiple group leaders were hence negligible, and there were no patterns indicating that any particular co-leader team inordinately affected findings.

### Session differences

Because there were differences in the length of sessions (not only between languages, but also within languages, reflected by the relatively large standard deviations for duration in those analyses), we analyzed total duration of coparenting events as a ratio of the total duration of each session. One-tailed Wilcoxon signed rank test found a marginally significant difference between Parenting and WDW sessions; groups averaged a higher percentage of time on coparenting events during WDW (Median = 8.84%, IQR [4.48, 15.68%]) than during Parenting sessions (Median = 4.97%, IQR [1.80, 10.21%]); z = −1.69, *p* < 0.05, r^2^ = 0.12, 95% CI [−0.01, 0.42]. Since sessions were presented in the same order to all groups, it is not possible to determine how much of this difference was a function of the respective topic of each session, and how much owed to an improving payoff in groups and group leaders effectively pursuing coparenting dialogues.

### Coparenting dialogues: differences among groups

A primary interest in this study was in examining the nature of coparenting events within couples groups, and so we asked whether any factors discriminated groups from one another on the basis of such events. K-means cluster analyses were used to identify groups with notably different patterns of such events. As there were relatively few numbers of coparenting events overall (M = 8.25, S = 5.40, range 1–20), we used the four main categories from the 10-level scale presented earlier: events that were “missed opportunities” (categories 1–2), events without meaningful payoff (3–5), events with minor payoff (6–7), and events with useful payoff (8–10). Given considerable variability in the total duration of sessions and total amount of time each group spent in all coparenting dialogue events, these variables were included in the cluster analysis. Because cluster analyses require standardized variables with normal distributions in order to reduce bias, we performed a square root transform on each variable with skewness > |0.80|, then converted all variables to z-scores.

A 2-group clustering solution offered a very simple picture. Cluster 1 had shorter average sessions and less total time in coparenting dialogue, plus less of each level of payoff (*p* < 0.01 in all cases) except total number of minor payoffs (*p* = 0.115). There were 13 couples groups in cluster 1 and 11 in cluster 2, with no statistically significant pattern of language across the clusters (χ^2^ [1, N = 24] = 2.59, *p* = 0.11, *φ* = 0.33). This presents the relatively unremarkable picture that shorter session length is associated with less coparenting dialogue.

However, a 3-group clustering solution offered a more intriguing picture. Table 2 shows the final cluster centers, with the alpha level of the contribution of each variable (all are statistically significant [*p* < 0.007] except for the total number of useful payoffs, which is marginally significant [*p* = 0.065]). In this model, cluster 1 (n = 9) had shorter average sessions, less total time in coparenting events, and fewer of all levels of payoff. Cluster 2 (*n* = 10) had the longest average sessions, most missed opportunities, and more of each other variable than cluster 1. It was cluster 3 (n = 5) that provided the intriguing addition to the 2-group model. This is a cluster of groups with session length times that ran less than cluster 2 but had much higher amounts of time discussing coparenting events. Moreover, though slightly above average in missed opportunities, cluster 3 also had a far greater number of all other levels of payoff (i.e., events without meaningful payoff, events with minor payoff, and events with useful payoff).

**Table 2:** Mean z-scores for the 3-group clustering solution, with *p*-values.

Variable (z-scores)	Cluster 1	Cluster 2	Cluster 3	*p-*value
Total time in sessions	−0.92	0.63	0.39	< 0.001
Total time in Coparenting events	−0.79	0.10	1.24	< 0.001
Total # missed opportunities	−0.85	0.69	0.14	= 0.001
Total # w/o meaningful payoff	−0.57	−0.03	1.08	= 0.006
Total # with minor payoff	−0.54	−0.15	1.28	= 0.001
Total # with useful payoff	−0.33	−0.15	0.90	= 0.065

Analyses examined were the total number of coparenting events; the number of group leaders’ initiating bids and responses to participants; the number of comments of wives as initiators, as respondents to husbands, as repeat commentators on their own issues within a bounded event, and as respondents to other group members; the comments of husbands as initiators, responders to wives, repeat commentators on their own issues, and respondents to other group members; and the responses of couples as a unit to other group members. Again, all skewed variables were normalized, then converted to z-scores. All analyses were BG ANOVAs with (2, 21) df.

The results were striking; of the 14 variables we examined as potential participant factors distinguishing among the clusters, all but three showed statistically significant differences among the three clusters. The three were: total number of husbands who responded to dialogues started by other couples (*F* = 1.795, *p* = 0.19), total number of responses by husbands to dialogues started by other couples (*F* = 1.037, *p* = 0.37), and total number of responses by wives during dialogues they themselves initiated (*F* = 2.878, *p* = 0.079). For the remaining variables examined, the three clusters did differ. Table 3 shows results for statistically significant BG ANOVAs of normalized, z-scored variables, with medians of raw scores on each variable for each cluster. We underscore the last column, which contains the median data from cluster 3 (relative to clusters 1 and 2).

**Table 3:** Statistically significant differences between clusters on normalized, standardized dependent variables, with medians of raw scores.

Variable	*F* (2,21)	*η^2^*	95% CI	Cluster 1	Cluster 2	Cluster 3
Total # events^c^ ‡	23.24***	0.69	0.38, 0.79	4	7	15
Total leader responses^c^ ‡	21.14***	0.67	0.35, 0.78	3	6	14
Total bids by leaders^a^	4.55*	0.30	<0.01, 0.51	2	1	4
# Initiated by husband^c^ ‡	11.25***	0.52	0.16, 0.67	1	3	7
# Initiated by wife^c^ ‡	10.39**	0.50	0.14, 0.66	3	5	10
Wife response as partner^c^ †	8.53**	0.45	0.09, 0.62	1	2	5
Husband response as partner^b^ ‡	9.13***	0.47	0.11, 0.64	1	2.5	6
Total # responses by husband to bids he himself initiated^b^ †	6.25**	0.37	0.04, 0.57	0	1	110
Total number of wives responding to others^b^ †	4.95*	0.32	0.01, 0.53	1	2	6
Total # responses of wives to other couples^b^ ‡	7.20**	0.41	0.06, 0.59	1	2	6
Total responses of H + W to other couples^b^ †	4.86*	0.32	0.01, 0.52	2	2.5	9

^a^ = this item is not correlated with either total time in sessions or total time of coparenting events (*p* > 0.10). ^b^ = these items are correlated (*p* < 0.05) with total time of coparenting events. ^c^ = these items are correlated with (*p* < 0.05) both total time in sessions and total time in coparenting events. * = *p* < 0.05; ** = *p* < 0.01; *** *p* < 0.001; † cluster 3 > cluster 2 = cluster 1; ‡ cluster 3 > cluster 2 > cluster 1; ‽ cluster 1 > cluster 2, cluster 1 = cluster 3.

The overall pattern was Cluster 3 > Cluster 2 > Cluster 1 on three of the four coparenting event categories – coparenting events without meaningful payoff, with minor payoff, and with useful payoff. It is hence perhaps not surprising that that same general ordering of the 3 clusters emerged for most variables in Table 2. Nonetheless, a few patterns that bucked this trend may hold interest. First, Cluster 3 had a higher average number of group leader comments (both as bids and on the dialogues of others). Second, husbands (but not wives) in Cluster 3 on average offered many more comments on dialogues they themselves had initiated. Clusters 1 and 2 were also equivalent (and worse than Cluster 3) in the frequency with which wives responded to their partner initiating an event and in the total number of responses by spouses to other couples who had initiated events. Otherwise, the analyses in Table 3 provide relatively little basis for drawing differences between Clusters 1 and 2.

## Discussion

Practitioners’ preparedness and capacity to scaffold deliberate exchanges between parents about coparenting and coparenting differences plays an important role in interventions aiming to improve communication, problem-solving and conflict resolution (see Figure 2). Yet specific clinical training in the detection and expansion of coparenting impasses, particularly in group settings, is uncommon. The aims of this study were to present a new strategy and coding approach to capture the essential nature of coparenting events within couples groups, attend to the inclinations of group leaders as potential influencers of these events, and explore how differences among the various groups studied may have captured greater or lesser success in elevating meaningful coparenting dialogues.

Somewhat surprisingly, the total overall number of coparenting events in relationship enhancement groups expressly conceived to address marital and parenting issues was relatively modest—we identified a total of 198 such events during the two specific sessions most closely relevant to coparenting across the 24 different couples groups analyzed. Approximately 6% of the overall session time analyzed contained coparenting events of any form. Events ranged from scenarios in which group leaders attempted to evoke a coparenting-related discussion but got no response from parents (who instead raised a different topic), to prolonged exchanges about coparenting involving multiple members of the groups. We compared English- and Spanish-speaking groups and though Spanish-speaking groups on average remained on topic for longer, there were no material differences in the proportion of group time allocated to coparenting events.

Although overall, there was not much coparenting discussion during the groups, data also indicated that coparenting conversations blossomed when group leaders got involved to help expand them. We note that in over a third of the instances identified (37%), higher-quality coparenting events (codes 8–10) materialized. Such instances often involved successful amplification of issues by group leaders, enabling a process that drew other members of the group to get involved. Both concrete coparenting prompts and frequency of participation by leaders once coparenting events were underway were important; indeed, over a third of all events (35%) were prompted by leaders. This finding suggests that the amount of session time spent on coparenting-related topics may have been even lower had it not been for such prompts.

From the perspective of practitioner training and supervision, focusing on both missed opportunities to amplify coparenting discussions (for example, in instances where parents’ comments are not responded to by their partners, or brief dialogues between partners that fail to catch the group’s and/or co-leaders’ attention from a coparenting point of view) and on more successful events (as when leaders’ amplification of issues allow other members of the group to get involved) afford opportunities for supervisors to help future practitioners develop greater attentiveness and preparedness to open dialogue. Specifically, supportive examination of coparenting events and of practitioners’ inclinations, successes and oversights during clinical training and supervision can promote increased mindfulness and ultimately lead to enhanced capacity for self-monitoring. We believe that such guided reflection, an important stepping stone in the training and professional competency building of practitioners who serve couples and families, can and should be more intentionally built into clinical training and continuing education programming.

Such an advance in clinical training stands to have significant impact. Unlike practitioners who conduct groups with individuals, those who lead couples groups must relate to the individuals and their interaction with leaders and other participants, while simultaneously dedicating special attention to the couple as a unit. This work is demanding and complex, as aptly detecting subtle instances of coparenting requires deliberate attunement by practitioners serving as group leaders, and preparedness to step in ably to capitalize on emergent coparenting events in couples groups to effectively amplify coparenting dialogues. Those who have developed both the intentionality and the skills for doing so will be better poised to help address important coparenting issues that entangle parents and, in some cases, adversely impact their children.

We believe the conceptual framework outlined above together with the scheme developed for tracking the progression of coparenting events once initiated will be helpful in advancing productive explorations of important coparenting issues in traditional couples group formats. Our experience watching the nearly 200 events described in this report leads us to advocate responding to burgeoning coparenting discussions using process-oriented rather than didactic approaches. Discussions are most likely to take off if leaders open dialogues between partners and take a position of guide or facilitator of the group process rather than teacher or expert. We look forward to future exposition and analyses of coparenting events within couple and relationship enhancement groups both to further advance coparenting theory and research, and to expand the training of future family practitioners.

## Data Availability

The raw data supporting the conclusions of this article will be made available by the authors, without undue reservation.

## Footnotes

^1^Analyses were conducted using SPSS/PASW 18.0. Where variables were normally distributed, statistical analyses were performed with relevant parametric tests (e.g., between- and within-group ANOVA, Pearson’s correlation). Where variables were skewed (skewness > |0.80|), appropriate non-parametric statistical analyses were performed (e.g., medians and inter-quartile ranges for univariate descriptive statistics, Mann–Whitney U for independent groups comparisons, Wilcoxon signed rank tests for dependent groups comparisons). Confidence intervals for correlations were computed using Fisher’s r-to-z transformation and using the exact method for η^2^ (c.f. Odgaard and Fowler, 2010).
